# Diabetes mellitus and the risk of glioma: a meta-analysis

**DOI:** 10.18632/oncotarget.6605

**Published:** 2015-12-14

**Authors:** Luqian Zhao, Zhiping Zheng, Ping Huang

**Affiliations:** ^1^ Department of Geriatrics, Guangzhou First People's Hospital, Guangzhou Medical University, Guangzhou 510180, Guangzhou, China

**Keywords:** diabetes, glioma, association, meta-analysis

## Abstract

Some studies reported a statistically significant inverse association between diabetes mellitus (DM) and risk of gliomas. However, the result is still controversial. We thus did a meta-analysis and summarized the evidence on the incidence of gliomas that has been studied in its association with DM. Seven case-control studies and 4 cohort studies were selected in this meta-analysis (n = 5898251). DM was significantly associated with decreased risk of gliomas (OR = 0.79; 95% CI 0.67 – 0.93; *P* = 0.004; *I^2^* = 59%). In the subgroup analysis of race, Caucasians of DM showed decreased risk of gliomas (OR = 0.81; 95% CI 0.69 – 0.94; *P* = 0.007). In the subgroup analysis of design, a statistically significant protective effect of DM on gliomas was observed in case-control studies (OR = 0.68; 95 % CI, 0.53–0.87; *P* = 0.002), while no such effect was observed in cohort studies (OR = 0.97; 95 % CI, 0.83–1.13; *P* = 0.70). In a further stratified analysis by gender, a significant association was found among males with DM (OR = 0.83; 95 % CI, 0.70–0.99; *P* = 0.04). No significant association was found between females with DM and gliomas (OR = 0.97; 95 % CI, 0.78–1.21; *P* = 0.81). In summary, this meta-analysis of current evidence suggests that DM is significantly associated with decreased gliomas risk in Caucasian and males.

## INTRODUCTION

Gliomas account for almost 80% of primary malignant brain tumors, and fewer than 50% of glioma patients live longer than 5 years after diagnosis [1]. Despite improvements in clinical care over the last 20 years, gliomas remain associated with considerable morbidity. To date, the only established environmental risk factor is exposure to moderate-to-high doses of ionizing radiation [2].

Diabetes mellitus (DM) is the most common endocrine disorder that affects 246 million people worldwide. The International Diabetes Federation (IDF) predicts that the number of people with DM will increase up to 380 million within twenty years [3]. Many studies suggest that DM is associated with an increased risk of cancer, such as liver, pancreas, endometrium, colorectum, breast, and bladder [4]. Recently, an umbrella review also found the significant associations between DM and risk of developing breast, cholangiocarcinoma (both intrahepatic and extrahepatic), colorectal, endometrial, and gallbladder cancer [5]. However, this study did not investigate the association between DM and risk of gliomas. Some studies reported a statistically significant inverse association between DM and risk of gliomas. However, the result is still controversial [6–16]. We thus did a meta-analysis and summarised the evidence on the incidence of gliomas that has been studied in its association with DM.

## RESULTS

### Characteristics of eligible studies

**Figure 1:**
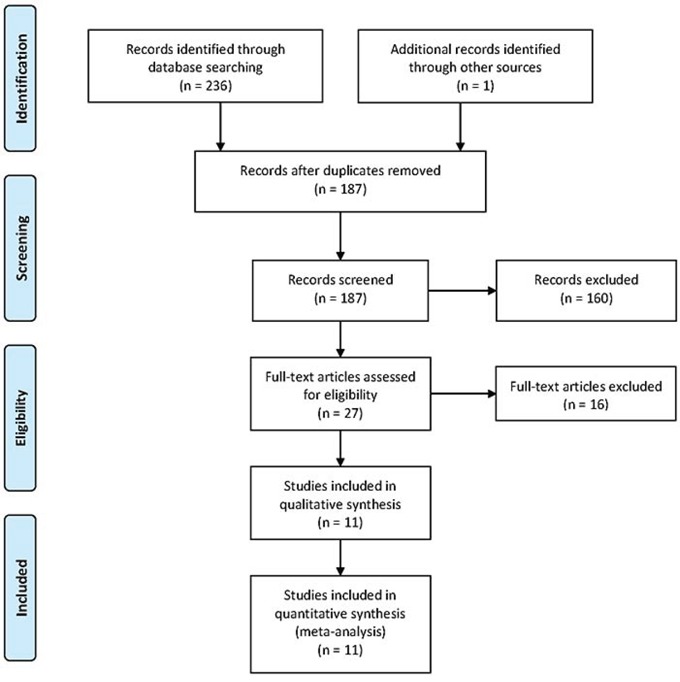
The selection of included studies

A total of 237 potential studies were identified by preliminary searching PubMed, Web of Science, Science Direct, EMBASE, and Cochrane Library databases, among which 7 case-control studies and 4 cohort studies were selected, involving a total of 5898251 subjects in this meta-analysis. The detailed literature search strategy was showed in Figure 1. The baseline characteristics, such as author name, publication year, ethnicity, design, age, gender, sample size, and covariants were depicted in Table 1. The quality of the 11 studies was high.

**Table 1:** Characteristics of the included studies

First author	Year	Study design	Race	Age	Male (%)	Sample size	Type of Diabetes	Covariant	NOS scores
Cicuttini	1997	Case-control	Caucasian	48.9	60	838	NA	Age and sex	8
Wideroff	1997	Cohort	Caucasian	64	50	109581	Mixed	NA	7
Schlehofer	1999	Case-control	Caucasian	20-80	54	3165	NA	Age and sex	8
Brenner	2002	Case-control	Mixed	18-90	57	1288	NA	Age, sex, race or ethnicity and distance of residence from hospital	8
Schwartzbaum	2005	Case-control	Caucasian	69	56	143573	NA	Age, sex, and year of diagnosis	8
Swerdlow	2005	Cohort	Caucasian	0-49	54	28900	I and II	NA	7
Stocks	2009	Cohort	Caucasian	44.8	61	30285	NA	Smoking, body mass index	8
Campbell	2012	Cohort	Mixed	>30	44	1053831	NA	Age, education, body mass index, smoking, alcohol intake, vegetable intake, red meat intake, physical activity, and aspirin use	9
Kitahara	2014	Case-control	Mixed	57	52	3157	NA	Age, sex	8
Cahoon	2014	Case-control	Mixed	52	100	4501578	NA	Age category, calendar time, race, and number of hospital visits.	9
Seliger	2015	Case-control	Caucasian	55.5	55	22055	NA	Age, sex, calendar time, general practice	8

NA, not available; NOS, Newcastle–Ottawa Scale.

### Association of DM and risk of gliomas

**Figure 2:**
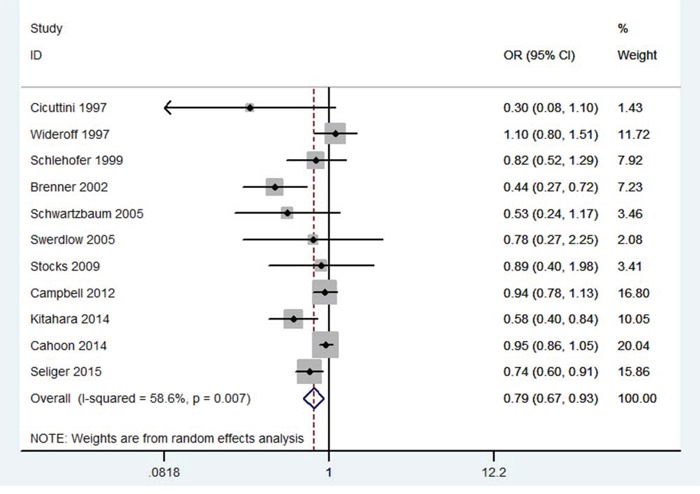
Meta-analysis of the association between DM and risk of gliomas

As shown in Figure 2, DM was significantly associated with decreased risk of gliomas (OR = 0.79; 95% CI 0.67 – 0.93; *P* = 0.004; *I^2^* = 59%). In the subgroup analysis of race, Caucasians of DM showed decreased risk of gliomas (OR = 0.81; 95% CI 0.69 – 0.94; *P* = 0.007). In the subgroup analysis of design, a statistically significant protective effect of DM on gliomas was observed in case-control studies (OR = 0.68; 95 % CI, 0.53–0.87; *P* = 0.002), while no such effect was observed in cohort studies (OR = 0.97; 95 % CI, 0.83–1.13; *P* = 0.70). In a further stratified analysis by gender, a significant association was found among males with DM (OR = 0.83; 95 % CI, 0.70–0.99; *P* = 0.04). No significant association was found between females with DM and gliomas (OR = 0.97; 95 % CI, 0.78–1.21; *P* = 0.81). The results were showed in Table 2.

**Table 2:** Meta-analysis results and subgroup analyses

	I^2^ (%)	Model	OR (95% CI)	*P* value
Overall	59	R	0.79 (0.67 – 0.93)	0.004
Caucasian	22	F	0.81 (0.69 – 0.94)	0.007
Case-control	72	R	0.68 (0.53 – 0.87)	0.002
Cohort	0	F	0.97 (0.83 – 1.13)	0.70
Male	70	R	0.83 (0.70 – 0.99)	0.04
Female	48	F	0.97 (0.78 – 1.21)	0.81

F, fixed effects model; R, random effects model.

**Figure 3:**
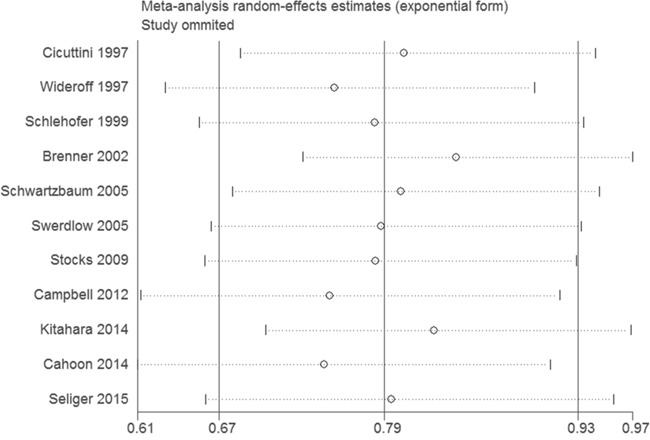
Sensitivity analysis of the association between DM and risk of gliomas

**Figure 4:**
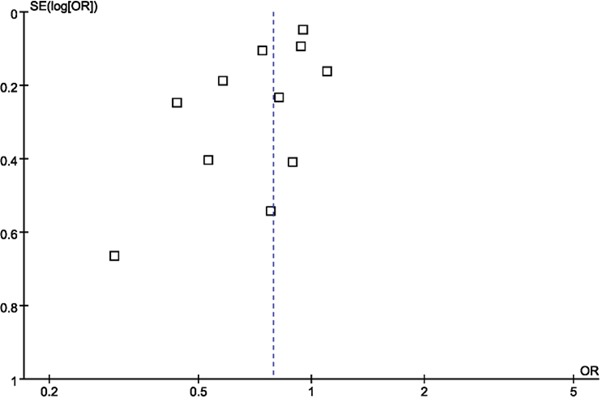
Funnel plot of the association between DM and risk of gliomas

Sensitivity analysis was carried out repeatedly by precluding a single study at a time. The results demonstrated that the estimates before and after the deletion of each study were similar (Figure 3). No evidence of publication bias was found in this meta-analysis by funnel plot (Figure 4) and Egger's test (*P* = 0.24).

## DISCUSSION

This is a comprehensive meta-analysis for clarification of the association between DM and risk of gliomas. Seven case-control studies and four cohort studies involving more than 580000 individuals were included in this study. The results suggested that DM was significantly associated with the decreased risk of gliomas. In the subgroup analysis by ethnicity, we noted that Caucasians with DM had decreased gliomas risk. However, there is no studies of Asians and other races. Although the pooled analysis from the case-control studies suggested a significant reduction in gliomas risk, the results from the cohort studies were non-significant, suggesting that our conclusion depend mainly on the case-control studies. It is generally thought that cohort studies provide stronger evidence regarding an association than case-control studies because they are less prone to recall or selection bias. In the subgroup analysis by gender, the inverse association between DM and gliomas was more apparent in men than women. Gender-specific hormonal changes in diabetic patients could serve as a possible explanation.

Previous studies suggested that DM might be a prognostic factor for cancers. Song et al. suggests that long-term DM is associated with an increased risk of pancreatic cancer [17]. Luo et al. found that DM was associated with an increased risk of colorectal neoplasia and adenoma [18]. Zhu et al. indicated that men with diabetes have a modestly increased risk of bladder cancer, while women with diabetes were not the case [19]. Chen and coworkers revealed that there was a significant negative impact of DM on overall survival (OS), cancer-specific survival (CSS), and recurrence-free survival (RFS) in renal cell carcinoma patients [20]. Zhou and coworkers revealed that women with DM are at higher risk of breast cancer [21].

Several limitations of this study should be noted. Firstly, only published studies that were included in the selected electronic databases were identified; it is possible that some relevant published or unpublished studies may have been missed. Secondly, no studies with Asians and other races was included in this meta-analysis. Thirdly, marked heterogeneity of studies was seen in this study. We attempted to find the exact factor that can account for the heterogeneity by subgroup analysis. Fortunately, the heterogeneity was decreased in the subgroup analysis of race. Fourthly, owing to the limited data in the included studies, we cannot analyze the associations between type 1 DM or type 2 DM and gliomas, separately. Finally, this study was meta-analysis of case-control study and cohort study. Confounding should be considered.

In summary, this meta-analysis of current evidence suggests that DM is significantly associated with decreased gliomas risk.

## MATERIALS AND METHODS

### Publication search

A systematic search was conducted in the PubMed, Web of Science, Science Direct, EMBASE, and Cochrane Library databases until Aug 16, 2015, with no limits. The search strategies were based on combinations of the following keywords: Gliomas, brain tumor, Diabetes mellitus, Diabetes, DM. The MeSH terms were glioma and diabetes mellitus. In addition, we checked relevant reviews on the topic of interest. We traced the reference lists of selected articles and used Google Scholar to find potential studies.

### Study selection

Studies were included in the meta-analysis if they fulfilled the following inclusion criteria: 1) study design: case-control or cohort studies; 2) population: DM patients; 3) primary outcome: the effect of DM on the risk of gliomas. Studies were included in the meta-analysis if they presented estimates of the OR or relative risk (RR) and the corresponding CI on the association between DM and risk of gliomas. When multiple reports were published on the same study population, we included the study with the largest number of cases. Abstract, case reports, review articles, experimental studies and commentary articles were excluded.

### Data extraction and qualitative assessment

Two investigators extracted data from the included studies independently, and the respective studies were retrieved for further consideration if judged pertinent by one or two reviewers. Any discrepancies were identified and resolved by consensus. For each study, the following data were extracted: first author's name, year of publication, study design, race, age, gender, sample size, and covariant. A modification of the Newcastle–Ottawa Scale (NOS) was used as an assessment tool for selection, comparability, and outcome assessment.

### Statistical analysis

OR and 95% CI were employed to evaluate the strength of the association between DM and risk of gliomas. The I^2^ statistic were used to assess the degree of heterogeneity among the studies included in the meta-analysis. If heterogeneity was observed among the studies (I^2^ >50%), the random-effects model was used to estimate the pooled OR (the DerSimonian and Laird method). Otherwise, the fixed-effects model was adopted (the Mantel–Haenszel method). Subgroup analyses were carried out by ethnicity, design, and gender. Sensitivity analysis was performed through sequentially excluded individual studies to assess the stability of the results. The potential publication bias was examined visually in a funnel plot of log [OR] against its standard error (SE), and the degree of asymmetry was tested using Egger's test. All statistical tests were performed using Revman 5.1 software (Nordic Cochrane Center, Copenhagen, Denmark) and STATA 11.0 software (Stata Corporation, College Station, TX, USA). A P value < 0.05 was considered statistically significant.
